# Technique and Outcomes of Radiofrequency Ablation of Biopsy-Proven 3–4 cm T1a Renal Cell Carcinoma

**DOI:** 10.3390/biomedicines13061296

**Published:** 2025-05-24

**Authors:** Mohamed E. Abdelsalam, Ahmed Awad, Roland L. Bassett, Thomas Lu, David Irwin, Ketan Y. Shah, Bruno C. Odisio, Peiman Habibollahi, Jose A. Karam, Surena F. Matin, Kamran Ahrar

**Affiliations:** 1 Department of Interventional Radiology, The University of Texas MD Anderson Cancer Center, 1515 Holcombe Boulevard, Houston, TX 77030, USA; 2 Department of Biostatistics, The University of Texas MD Anderson Cancer Center, Houston, TX 77030, USA; 3 Department of Urology, The University of Texas MD Anderson Cancer Center, Houston, TX 77030, USA

**Keywords:** technique, ablation, outcomes, survival rates

## Abstract

**Objective:** The American Urological Association recommends ablation as an alternative treatment option for T1a RCC smaller than 3 cm. Our objective is to describe our technique and evaluate the outcomes of radiofrequency ablation (RFA) for biopsy-proven T1a RCC measuring 3–4 cm, compared to outcomes for tumors <3 cm. **Materials and Methods:** A single-center, retrospective review included patients with solitary, de novo, biopsy-proven T1a RCC who underwent RFA between January 2001 and December 2020. Using propensity score matching, patients with 3–4 cm lesions (Group A) were matched with patients with lesions less than 3 cm (Group B) based on the pathology, grade, duration of follow-up, another primary malignancy, age, and sex. Survival outcomes were estimated using the Kaplan and Meier product-limit estimator, and both groups were compared. **Results:** A total of 122 patients were included in the matched analyses. Eight patients were missing data on disease recurrence, leaving 114 patients with data on RFS and DFS (55 patients in Group A and 59 patients in Group B). The median tumor size in groups A and B was 3.3 cm and 2.2 cm, respectively. There was no statistically significant difference in the complication rate (*p* = 0.11) and local recurrence at the ablation site (*p* = 0.15). There was no statistically significant difference in overall survival (*p* = 0.93), recurrence-free survival (*p* = 0.45), or disease-free survival (*p* = 0.37). The metastasis-free survival and cancer-specific survival were 100% in both groups. **Conclusions:** RFA is a highly effective modality for the treatment of 3–4 cm T1a RCC, with long-term favorable oncologic and survival outcomes.

## 1. Introduction

The critical need to preserve kidney function has led to growing interest in minimally invasive procedures for smaller renal masses [1,2,3,4,5,6]. The American Urological Association (AUA) advocates for partial nephrectomy when treating these masses, leaving traditional radical nephrectomy for specific cases [6]. Some patients, however, are either not suitable for surgery or prefer not to undergo it due to potential perioperative risks. For such individuals, percutaneous image-guided thermal ablation (TA) has become an appealing alternative [6,7,8,9]. It is especially crucial for those with multiple renal tumors, those who are at risk of or have chronic kidney disease, or those who have syndromes like Von Hippel Lindau that increase the risk of renal cell carcinoma (RCC) to preserve kidney function. TA not only offers local control over cancer in a nephron-sparing fashion but also comes with reduced complications, quicker recovery, and the potential for outpatient treatment compared to surgical options [5]. A growing body of evidence showcasing the oncological efficacy and survival outcomes of thermal ablation has gained recognition from various professional societies [6,8,9,10]. As of 2017, the AUA has acknowledged thermal ablation as a viable treatment option for lesions smaller than 3 cm in select patients [2,6]

Introduced in the 1990s, radiofrequency ablation (RFA) became a treatment option for RCC patients who are not candidates for extirpative surgery. The literature detailing its efficacy, along with its short- and mid-term results, has since expanded [2,11,12,13,14,15,16,17,18,19,20], as well as studies describing its long-term outcomes spanning more than five years [21,22,23,24,25].

The purpose of this study is to describe our RFA technique and to evaluate its oncological and survival outcomes for patients with de novo, solitary, biopsy-proven T1a RCC 3–4 cm in size compared to outcomes regarding tumors < 3 cm.

## 2. Materials and Methods

We conducted a retrospective review of all patients (n = 860) listed in our institutional renal ablation registry from January 2001 to December 2020 and identified all the patients with T1a RCC (up to 4 cm). Our study received approval from the Institutional Review Board, and the requirement for informed consent was waived. We included patients who had a solitary, primary cT1aRCC 3 cm or larger in size, confirmed through tissue biopsy, and who had undergone percutaneous image-guided radiofrequency ablation. We excluded individuals with syndromes that increase RCC risk, such as Von Hippel Lindau syndrome, those with a prior personal history of RCC, those with RCC metastasis, those presenting with multiple renal masses, and patients with benign tumors or those without biopsy-confirmed RCC. To ensure a precise evaluation of outcomes for patients with T1a RCC lesions of 3 cm or larger (Group A), we utilized propensity score matching, based on pathology, grade, follow-up duration, presence of another primary non-renal malignancy, age, and gender, to create an equivalent number of patients with RCC lesions under 3 cm (Group B).

### 2.1. Ablation Technique

**Figure 1:**
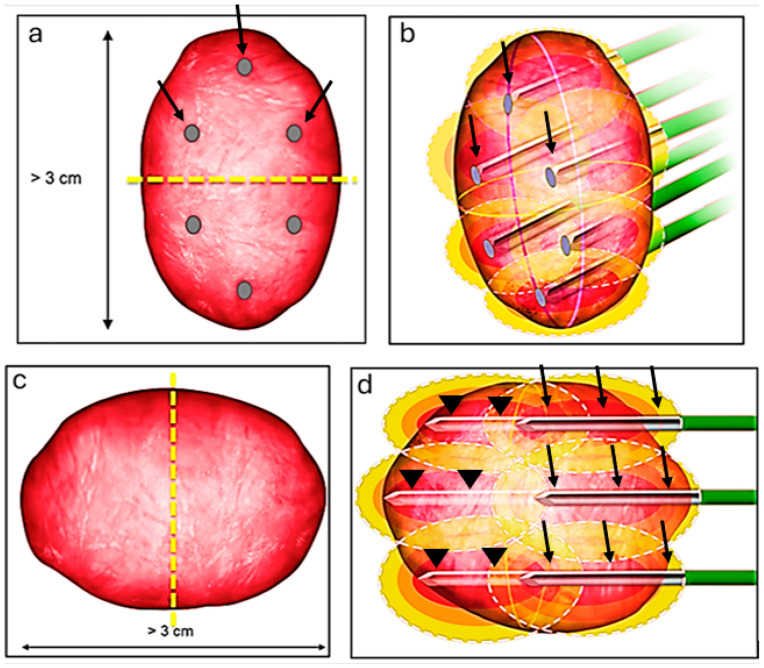
Ablation technique for lesions larger than 3 cm. The craniocaudal size is the dominant dimension (larger than 3); we bisect the tumor at its center (yellow dash line) and strategize the ablation as though handling two distinct tumors (**a**). Initially, we address the cranial part using 3 probes (black arrows), then reposition the 3 probes to target the more inferior part (**b**). If the tumor’s width (transverse dimension) is the greater dimension, we again divide the lesion at its center (yellow dash line) (**c**). First, we position the 3 probes (arrow heads) to address the deeper portion of the tumor and subsequently retract the probes (black arrows) to treat the superficial portion of the tumor (**d**).

We have detailed our ablation technique in prior publications [7,26]. In summary, we perform the RFA procedures with patients under general anesthesia, guided by computed tomography (CT) imaging (specifically, SOMATOM Definition AS from Siemens Medical Systems, Erlangen, Germany). Once we have planned the ablation, we position the probes and conduct a biopsy (if it has not been carried out beforehand) before initiating the ablation. We use the Cool-tip RF system (Covidien, Mansfield, MA, USA) and employ adjunctive techniques like hydrodissection and pyeloperfusion as necessary. For tumors less than 3 cm, we use 3 cm active tip probes (typically N + 1, where N represents the tumor’s size), positioning them within the tumor. Larger tumors typically require more intricate planning to ensure complete ablation of the tumor. If the craniocaudal size is the dominant dimension (larger than 3), we bisect the tumor at its center and strategize the ablation as though handling two distinct tumors (Figure 1a). Initially, we address the cranial or the caudal part of the tumor using 3 probes, then reposition the 3 probes to target the other part of the tumor (Figure 1b). If the tumor’s width (transverse dimension) is the greater dimension, we again divide the lesion at its center (Figure 1c). First, we position the 3 probes to address the deeper portion of the tumor, and subsequently retract the probes to treat the superficial portion of the tumor (Figure 1d).

**Figure 2:**
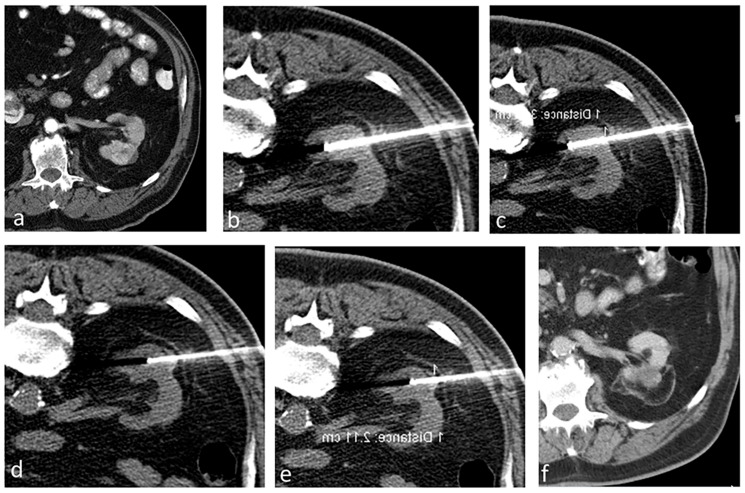
A patient with a 3.9 cm biopsy-proven RCC treated using RFA. (**a**) Contrast-enhanced CT showing a renal mass (3.9 cm in transverse diameter) in the left kidney. (**b**–**e**) Ablation procedure. (**b**,**c**) Non-contrast axial CT showing the probes positioned to treat the deeper portion of the tumor, and subsequently the probes were retracted to treat the superficial portion of the tumor (**d**,**e**). (**f**) Contrast-enhanced CT after 5 years showing no evidence of residual or recurrent tumor in the left kidney.

Immediately after the ablation, we carry out a multi-phase contrast-enhanced CT to evaluate the ablation area and detect any immediate complications. Thereafter, we schedule follow-up imaging at regular intervals for a period of 2 years, followed by annual checks. Figure 2 shows a case in the study that has a transverse diameter of 3.9 cm treated by the aforementioned technique.

### 2.2. Data Collection

We perused each patient’s electronic medical record and gathered the following details: demographic information (including age and sex), renal tumor characteristics (size and side), tumor histology (specific type and Fuhrman grade), any history of other non-renal cancers, technical success of the ablation, the technology used in thermal ablation, the type of imaging used for guidance, any adjunctive techniques, complications (classified by the Clavien–Dindo system), any residual disease or recurrence at the ablation site, tumor recurrence elsewhere in the kidney (outside the ablation zone), development of RCC metastases post ablation, the patient’s current living status, and if applicable, the date and cause of death.

### 2.3. Definitions of Outcomes

A residual tumor is defined as any contrast enhancement observed within the ablation zone during the first post-procedure follow-up cross-sectional imaging. Tumor recurrence refers to any newfound contrast enhancement within or at the edges of the ablation area not spotted in first follow-up imaging or the detection of live tumor cells within the ablation zone upon tissue biopsy. Both residual and recurrent tumors were evaluated for potential surgery, active surveillance, or another ablation session.

We base our survival outcome definitions on the AUA guidelines pertaining to small renal mass management [3]. Overall survival (OS) denotes the probability that patients were still alive at the last follow-up assessment. Local recurrence-free survival (LRFS) reflects the probability that patients remained alive without any recurring tumors in the ablation area. The metastasis-free survival (MFS) was defined as the probability that patients did not develop RCC metastases in remote organs. Disease-free survival (DFS) represents the probability that patients remained alive with no findings of RCC disease in the ablation zone, elsewhere in the kidneys (outside the ablation zone), or in a distant organ on the last imaging. Cancer-specific survival (CSS) portrays the probability that patients did not die from RCC.

### 2.4. Data Analysis and Statistics

We used descriptive statistics to present all demographic data, tumor characteristics, ablation procedure details, and pathology results. The Kaplan–Meier method was used to estimate the distributions of OS, LRFS, DFS, MFS, and CSS. These survival rates were calculated starting from the ablation procedure date up to the date of diagnosing a recurrence, either within the ablation area or other parts of the kidney, the development of metastasis, or the event of death. The oncologic and the survival outcomes were compared between both groups.

## 3. Results

In our matched analyses, 122 patients were included (84 males and 38 females, with a median age of 68.7 years) and distributed evenly (61 in each group). Of these, 8 lacked follow-up imaging, leaving 114 patients for LRFS and DFS evaluation (55 from Group A and 59 from Group B). Among them, 67 (55%) had another primary cancer, with 32 from Group A and 35 from Group B (*p* = 0.917). Table 1 elaborates on the demographics and tumor characteristics.

### 3.1. Procedural Results

We conducted 122 ablation treatments for 122 renal tumors across the 122 participants. The right kidney harbored 77 (or 63%) of the tumors, while the remaining were in the left. CT-guided RFA was used in all procedures. Every procedure achieved technical success in terms of successful probe placement and performing TA. Median tumor sizes for Group A and B were 3.3 cm (range: 3–4 cm) and 2.2 cm (range: 1.3–2.9 cm), respectively. Out of 122 patients, a total of 9 (7.3%) patients developed complications, including both minor and major complications; there was not a significant difference in complication rate between both groups (*p* = 0.11). Table 2 summarizes the complications, including their classification based on the Clavien–Dindo grading system and the corresponding management strategies.

### 3.2. Pathological Results

Biopsies of the 122 tumors revealed different histological subtypes, predominantly the clear cell RCC subtype (89%) and papillary RCC (9%). The majority exhibited a Fuhrman grade of 2 (77.9%). Table 1 presents the pathology variants and Fuhrman grading.

### 3.3. Oncologic Outcomes

All patients had clinical and radiological follow-up by the Urology and Interventional Radiology teams. Eight patients did not have any follow-up imaging after the ablation procedure and were excluded from the analysis. The median OS for the whole patient population in this study was 7.3 years.

#### 3.3.1. Residual Disease or Local Recurrence

Among the 114 patients, none showed residual disease during the follow-up period. Local recurrence was detected in five patients (4.3%): four from Group A and one from Group B. There was no significant difference in recurrence rates between both groups (*p* = 0.15). All recurrences were clear cell RCC and were noticed after a minimum 6-month interval, with a median detection time of 10.3 months (range: 6–26.2 months). All patients were managed either by repeat thermal ablation (n = 3), partial nephrectomy (n = 1), or active surveillance (n = 1).

#### 3.3.2. Disease Recurrence and Distant Metastases

Nine participants (7.8%) had tumor recurrence in the kidney outside the ablation zone, with a median detection time of 52.5 months. Recurrence rates between the groups did not significantly vary (*p* = 0.23). One of these patients underwent thermal ablation, while the other eight received active surveillance. None of the patients developed metastatic disease from RCC at the time of data collection; therefore, the MFSwas 100%.

### 3.4. Survival Outcomes

**Figure 3:**
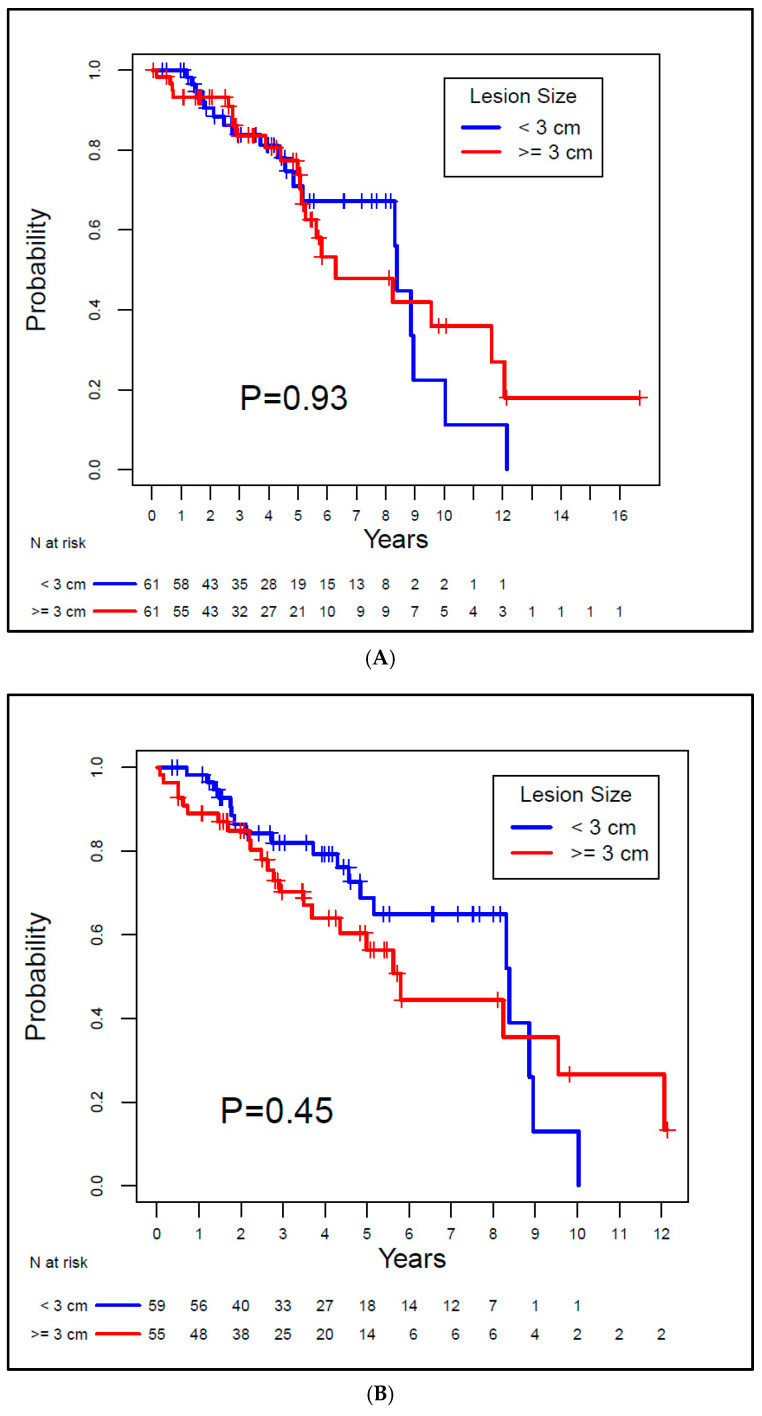

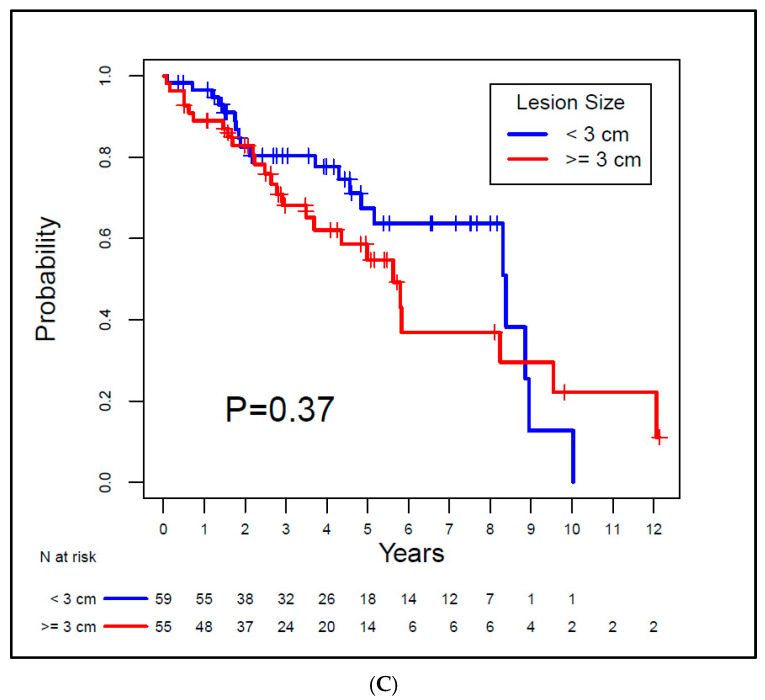
Kaplan–Meier overall survival (**A**), local recurrence-free survival (**B**), and disease-free survival (**C**) curves for the study patients.

The median OS for Group A was 8.39 years, and for Group B, it was 6.29 years. There was no evidence of a significant difference between the groups (*p* = 0.93). For Group A and Group B, the median LRFS was 8.39 years and 5.8 years, respectively, also with no evidence of a difference between groups (*p* = 0.45). The median DFS for the groups was 8.39 years and 5.62 years, respectively, with no statistically significant difference between groups (*p* = 0.37). Both MFS and CSS rates were 100% for both groups since none of the patients exhibited metastatic disease or died from RCC. Kaplan–Meier curves for OS, LRFS, and DFS are depicted in Figure 3.

## 4. Discussion

This study demonstrates that the oncological and survival results of RFA for T1a RCC lesions 3 cm or larger (Group A) in size are comparable to those for lesions smaller than 3 cm (Group B). Our findings reveal no discernible difference in recurrence rates between tumors under 3 cm and those 3 cm or larger. These findings are in concordance with the local oncologic outcomes by Zagoria et al. [19]. Even though they noted five recurrences (12%), all were in lesions exceeding 4 cm. No recurrences were observed in lesions under 4 cm (T1aRCC) [19].

However, our oncological and survival outcomes differ from those reported by Johnson et al. [22]. When they categorized tumors based on size (under 3 cm versus 3 cm or larger), a significant difference was evident in recurrence and survival rates. The authors reported a significant decline in the 6-year OS from 97% to 68% for lesions 3 cm or larger. Similarly, a significant decrease was seen in both LRFS and DFS [22].

In our study, we found no discernible differences in recurrence or survival rates between the two groups: those with cT1a tumors equal to or larger than 3 cm and those with tumors smaller than 3 cm. The differences in outcomes between our study and the one conducted by Johnson et al. could stem from several factors. First and foremost, the dissimilarities could potentially be attributed to variances in the technical aspects of the ablation procedures employed. Johnson et al. utilized multitined electrodes (StarBurst XL, Rita RFA System) and performed two ablation cycles for each tumor. In approximately 24.1% of cases, they employed a laparoscopic approach with ultrasound guidance. By contrast, our approach significantly differs from that of Johnson et al. For the RFA of renal tumors, we utilize Cool Tip and perform multiple overlapping ablations, which are tailored to the size and orientation of the tumor. All the patients in our study underwent percutaneous procedures under the guidance of CT. Additionally, a contrast-enhanced CT scan was conducted at the conclusion of each procedure to confirm the complete ablation of the tumor. If the CT images revealed any residual disease or inadequate safety margins, we performed additional overlapping ablations to minimize the risk of recurrence by ensuring complete tumor ablation with adequate safety margins. Secondly, only 55% of the cases in the study by Johnson et al. had biopsy-confirmed renal cell carcinoma (RCC), potentially raising the possibility that some of the smaller lesions they treated might have been benign lesions. By contrast, all cases in our study were confirmed to be RCC through biopsy. Thirdly, Johnson et al. included patients with multiple tumors or a previous history of RCC, whereas our research exclusively focused on individuals with a single, newly developed RCC lesion. Lastly, while we exclusively studied T1a patients, Johnson et al. included both T1a and T1b cases in their research.

Our results of RFA of T1a RCC are in concordance with the results published by other investigators. Psutka et al. [23] discussed their RFA outcomes for T1a and T1b RCC lesions. In their T1a RCC subgroup, they reported a 5-year local RFS of 96.1% and a 10-year local RFS of 93.2%. These findings align closely with our study’s local RFS rates of 98.2% for Group A and 91.5% for Group B at 5 and 10 years. Additionally, they observed a 5-year DFS of 91.5% [23], whereas our research showed 5-year DFS rates of 94.1% and 89.3% for Groups A and B, respectively. Furthermore, our 100% MFS and CSS rates echo their published rates [23]. Similarly, our results are in alignment with long-term outcomes of laparoscopic partial nephrectomy for T1a renal masses. A retrospective analysis conducted by Brassetti et al. [27] at a single center evaluated the long-term oncologic outcomes of purely off-clamp laparoscopic partial nephrectomy in 63 patients with a median tumor size of 3 cm. The study reported a median follow-up of 171 months. At 15 years, the DFS, CSS, and OS rates were 68%, 90%, and 72%, respectively. The rates of local recurrence and distant metastases were 2% and 17%, respectively [27].

A strength of this study is the well-matched patient cohort, which reaffirms the reliability of the results. Another strength is including only biopsy-proven RCC cases, thus eliminating any potential bias secondary to including patients with benign lesions. A limitation of this study is its retrospective design with all the inherent limitations, e.g., patient selection bias, which can potentially impact the validity and relevance of the results. A prospective randomized trial would eliminate the selection bias and increase the power of the study. Another limitation is the small sample size. A bigger sample size with a longer follow-up duration would reveal more precise estimations of survival outcomes for both groups. Furthermore, the current study reflects a single-institution experience; the generalizability of the results should be interpreted with caution. A multi-institutional study with a standardized ablation technique and a larger, more representative patient population would enhance the research validity and applicability of the findings.

## 5. Conclusions

In summary, RFA is an efficacious therapeutic option for patients with T1a RCC equal to or greater than 3 cm. Long-term data reveal favorable oncologic control and survival outcomes comparable to those of lesions less than 3 cm. The current recommendations of the AUA guidelines for thermal ablation of lesions up to 3 cm may warrant a re-evaluation contingent upon the emergence of further supporting data.

## Figures and Tables

**Table 1:** Patients and tumor characteristics (n = 122).

Characteristic	Group A ≥ 3 cm		Group B < 3 cm		*p* Value
	n = 61	%	n = 61	%	
Age, years					**0.60**
Mean (SD)	67.5 (11.3)		69.5 (10.6)		
Median (range)	68.4 (28.6–85.3)		70.6 (49.5–89.4)		
Gender					**1.00**
Female	19	31	19	31	
Male	42	69	42	69	
Another primary malignancy					**0.72**
Yes	32	52.5	35	57.4	
No	29	47.5	26	42.6	
Size of lesion, cm					
Mean (SD)	3.3 (0.3)		2.1 (0.4)		
Median (range)	3.3 (3.0–4.0)		2.2 (1.3–2.9)		
Kidney Laterality					**1.00**
Left	22	36.1	23	37.7	
Right	39	63.9	38	62.3	
Ablation modality					**1.00**
RFA	61	100	61	100	
Guidance modality					
CT	61	100	61	100	**1.00**
Pathology					**1.00**
RCC, Clear cell	55	90.2	54	88.5	
RCC, Papillary	5	8.2	6	9.8	
RCC, Not otherwise specified	1	1.6	1	1.6	
Grade					**1.00**
1	8	13.1	8	13.1	
2	47	77	48	78.7	
3	6	9.9	5	8.2	

**Table 2:** Complications, Clavien–Dindo grade, and their management.

Complication	Clavian-Dindo Grade	Management
Urinoma	Grade I	Conservative (observation)
Retroperitoneal hematoma	Grade III	Angiography and embolization
Pneumothorax	Grade III	Chest tube
Pneumothorax	Grade I	Conservative (observation)
Perirenal hematoma	Grade II	Blood transfusion
Hematuria, obstructing clot	Grade III	Percutaneous nephroureteral catheter
Subcapsular hematoma	Grade I	Conservative (observation)
Subcapsular hematoma	Grad III	Angiography and embolization
Subcapsular hematoma	Grade I	Conservative

## Data Availability

The datasets generated and/or analyzed during the current study are available from the corresponding author upon reasonable request.

## List of Abbreviations

AUA	American Urological Association
TA	Thermal ablation
RCC	Renal cell carcinoma
RFA	Radiofrequency ablation
CT	Computed tomography
OS	Overall survival
LRFS	Local recurrence–free survival
MFS	Metastasis-free survival
DFS	Disease-free survival
CSS	Cancer-specific survival
